# Study protocol: watchful observation of patients with limited small cell lung cancer instead of the PCI—prospective, multi-center one-arm study

**DOI:** 10.1186/s12885-020-06721-8

**Published:** 2020-03-18

**Authors:** Sergiusz Nawrocki, Anna Sugajska

**Affiliations:** Katedra Onkologii, Wydział Lekarski, Collegium Medicum, Uniwersytet Warmińsko-Mazurski w Olsztynie, Olsztyn, Poland

**Keywords:** Small-cell lung cancer, Prophylactic cranial irradiation, PCI, Stereotactic radiotherapy, SRT

## Abstract

**Background:**

Prophylactic cranial irradiation (PCI) is a current standard of care after confirmed response to radical chemoradiotherapy for limited disease small cell lung cancer (LD-SCLC). This standard is mostly based on results of old randomized studies when brain imaging with magnetic resonance (MRI) was not available. Survival benefit of PCI in extended disease SCLC was recently challenged by the results of randomized phase III study from Japan.

**Methods:**

Eighty patients with LD-SCLC after response to chest chemoradiotherapy will be enrolled. Patients will be followed up by brain MRI every 3 to 6 months up to 3 years. Neurocognitive function tests will be performed at baseline and after 12 and 24 months. Patients who develop brain metastases will be irradiated with stereotactic (SRT) or whole brain RT (WBRT). The primary endpoint is overall survival. The secondary endpoints are: response rate to radiotherapy of early detected brain metastases, analysis of efficacy of SRT and WBRT; assessment and analysis of neurocognitive functions and QoL in the studied cohorts: QLQ-C30 questionnaire and the California Verbal Learning Test, Color connection test, Benton visual retention test, and verbal fluency test will be carried out.

**Discussion:**

The results of this trial may contribute to changing of LD-SCLC clinical management by deescalating the treatment. There is a lack of prospective, recent studies in LD-SCLC patients with omission of PCI and modern radiation therapy technologies for developed brain metastases. The comprehensive neurocognitive function testing will help to assess the impact of modern radiotherapy (SRT) compared with WBRT and no-PCI in SCLC patients. A subgroup of long-term survivors, who will not develop brain metastases, will not be exposed to unnecessary brain irradiation with its deleterious consequences. The limitation of our study is a lack of parallel randomized control arm. This is a potential source of bias; however, randomized study will be difficult to complete for two major reasons: (1) limited population of LD-SCLC eligible for the study and (2) opinions of our patients, who after information and discussion about benefits and potential harms of PCI, often choose to omit PCI in our practice.

**Trial registration:**

ClinicalTrials.gov Identifier: NCT04168281, 19 Nov. 2019.

## Background

About 15% of patients with lung cancer are diagnosed with SCLC, one third of whom are patients with LD. According to the current standard clinical practice, patients with known LD-SCLC after a radical treatment (chemotherapy and chest radiotherapy) are eligible for prophylactic cranial irradiation (PCI) in case of a good response to treatment and lack of CNS dissemination. PCI aims to reduce the risk of brain metastases development and to improve survival, however, at the expense of possible losses of cognitive functions (attention, perception, memory, executive functions) because of brain irradiation.

This wide clinical acceptance of PCI is based mainly on the results of the meta-analysis published in 1999, which summarizes the observations of patients treated in the 70’s and 80’s [1]. At that time, the staging of the patient’s disease was mostly based on X-ray, biopsy and computed tomography (CT) scans were not performed. The treatment of patients was varied, and the methods (especially radiotherapy) differed significantly from those used nowadays. Three years after randomization, cerebral metastases occurred among 60% of patients in the non-PCI group, whereas only 40% of the patients in the PCI group were diagnosed with metastases to the brain. Based on the results of this meta-analysis, it has been demonstrated that there is a reduction in the risk of brain metastases by 20% after PCI, and an overall survival improvement of about 5% was observed. The patients analyzed in the meta analysis formed a heterogeneous group, which is difficult to compare with patients treated today mainly due to the phenomenon of stage migration. Certainly, in the prophylactically treated PCI cohort, there were some patients with already developed brain metastases.

In 2007, the results of a prospective study evaluating the influence of PCI on the survival and progression of the disease in the group of patients with disseminated small cell lung cancer were published [2]. Similarly, the irradiated group was compared with the group without PCI. It has been shown that after PCI treatment, there is a 2-time reduction in the risk of symptomatic metastases to the brain and an improvement in overall survival.

The introduction of CT and, more importantly, MRI as methods of brain metastases imaging in the last two decades has important implications for interpretation of results of trials with PCI. Even in the era of the common use of CT of the brain in LD SCLC, if in 90% of the cases there were no detected metastases, 6% single metastasis was found and 4% multiple metastases were found when CT was used, whereas in the same group of patients evaluated with the MRI of the brain, 76% were observed as no metastases, 14% were seen with multiple metastases, and 10% for single metastasis [3]. This comparison of CT and MRI sensitivity in SCLC suggests that many patients treated with PCI in pre-MRI era have had asymptomatic metastases to the brain so that their irradiation was indeed not prophylactic but selective which might influence the reported results upon which current clinical guidelines are based.

Recently, in Japan, a randomized, 3-phase study was performed using MRI imaging [4] in a group with disseminated SCLC (ED-SCLC), in which the PCI in the experimental arm was replaced with watchful observation with regular MRI follow-up. In the group closely followed up without PCI, MRI was performed at regular intervals. PCI reduced the number of diagnosed brain metastases by about 20% in the 24-month follow-up. At the same time, it was demonstrated that PCI does not improve the overall survival of patients. On the contrary, PCI could even have had negative effect on survival (HR = 1,27; statistically non-significant). It could be that patients qualified for PCI experienced significant neurotoxicity of treatment, deterioration of cognitive functioning [5], which might contribute to non-cancer deaths. This study confirmed the belief prevailing in Asian countries about avoiding the PCI in standard treatment of SCLC.

In conclusion, the standard of treatment for patients after radical treatment of small-cell lung cancer is a consequence of research conducted before universal access to MRI, and MRI examination reveals much more metastatic changes to the brain than CT.

To our knowledge, no prospective studies using current diagnostic and therapeutic options for patients with LD small-cell lung cancer have been performed assessing the omission of PCI. In recent years, two retrospective studies evaluating the group of patients in the localized stage have been published. In the Ozawa study in 2015 [6], it was shown that performing PCI in the group of LD SCLC patients may bring questionable benefit in the situation when there is wide access to MRI research, and at the time of recurrence it is possible to perform SRT. A 2016 study [7] has shown that more research is needed to detect patient group with no benefits from PCI. Probably, these are patients older than 70 years and with a tumor larger than 5 cm in the lung. In clinical practice, these criteria are met by most our patients.

Our trial is aimed to fill this gap and to answer several important questions regarding the need of PCI in LD-SCLC in current clinical practice.

Brain irradiation is associated with both acute and chronic toxicity. Acute toxicity associated with PCI is fatigue, alopecia, redness of the scalp, and to a lesser extent, headache and nausea of low intensity. All of these are self-limited [8]. Long-term toxicity, especially delayed neurocognitive disorders are important especially in the group of LD patients with long survival or cure. Significant neurological and intellectual deficits were observed historically when brain irradiation was performed with simultaneous chemotherapy, in high fractions (3.0 or 4.0 Gy), or to a high total dose, all of which have been shown to be associated with significant late neurotoxicity [9–11].

The probability of severe deficits appears to be much lower with the use of modern irradiation techniques, lower fractions, and total doses. Two historical randomized studies monitored patients after PCI. In the first trial, patients underwent a neuropsychological examination and CT of the head, which were repeated during the follow-up in the group of patients with and without PCI (total dose 24 Gy in 2-Gy daily fractions) [12]. No significant differences in neuropsychological functioning have been described. In the second trial, the cognitive functions and QoL of patients were evaluated both before and after the treatment with the PCI and in the control group [13]. None of these studies showed statistically important PCI-related adverse events; however, the treatment was performed before MRI era so the relevance of these historical results today is limited.

More recently, RTOG 0212 study evaluated a group of 265 patients with LD-SCLC after therapy which included PCI with different total dose and fractionations (25 Gy in ten fractions vs 36 Gy in 2 Gy daily or 24 twice-daily fractions) [14]. A detailed neurocognitive and quality of life assessment was conducted in the studied population. The initial assessment before PCI detected abnormalities in many parameters (language, visual-spatial scan, attention, sequencing, and quickness). The progression of neurological dysfunction was observed in the 12-month evaluation, but chronic neurotoxicity was less common in patients treated with a lower dose (60 vs. 85–89%, *p* = 0.02).

As the treatment of SCLC will hopefully become more and more effective so the potential neurotoxicity of PCI should be minimized or better eliminated in patients who would never develop brain metastases. Research efforts aimed at reducing the neurotoxicity of PCI included, among others, hyperfractionation (1.5 Gy 2 × daily to 30–36 Gy), WBRT with hippocampal sparing, as well as the use of pharmacological methods (cytoprotection), such as memantine [15, 16]. Trials with hippocampal sparing and memantine were small and underpowered so that these methods have not been widely accepted in clinical practice.

The aim of our study is to limit the irradiation of the brain to a group of patients who develop metastases to the brain. The primary end-point of the trial is the assessment of patients’ overall survival at 24 months after starting radical treatment with chemoradiotherapy. We assume that careful follow-up with regular MRI and the use of modern irradiation technologies in case of diagnosed early brain metastases will not worsen survival in LD-SCLC patients without PCI. The second primary endpoint is intracranial tumor control at 12 and 24 months after patient recruitment.

The secondary goals are (1) to assess the risk of developing brain metastases without PCI; (2) to assess the efficacy of radiotherapy of detected early brain metastases, including the feasibility and efficacy of SRT (stereotactic radiotherapy); (3) to monitor patients with neurocognitive tests and QoL questionnaires to asses and compare the influence of treatment on their performance and QoL in subgroups.

## Methods/design

Patients diagnosed with LD-SCLC after completed radical treatment (chemoradiotherapy according to current international clinical guidelines) will be eligible for the study (Table 1). Eligible patients should have brain MRI at initial diagnosis with contrast before chemoradiotherapy and after completion of chemoradiotherapy. MRI studies will be acquired on a 1.5-T scanner using a dedicated coil for brain imaging using standard brain imaging MRI protocol: T1-, T2-, diffusion- weighted, and fluid-attenuated inversion recovery (FLAIR)], followed by T1-GD images.

**Table 1:** Major eligibility criteria

Inclusion criteria	Exclusion criteria
Diagnosis of LD-SCLC	Lack of signed informed consent of the patient
A radical treatment (chemoradiotherapy according to current clinical guidelines) with remission or good/very good response to treatment	Disseminated neoplastic disease
Good general condition of the patient (ECOG/WHO − 0-2)	Contraindications for cerebral MRI examination
MRI of the brain in staging before chemoradiation treatment	The patient is not able to perform tests assessing cognitive functions
Signed informed consent	Poor performance status (ECOG/WHO > 2)

The eligibility criteria and doses for SRT: 1–3 asymptomatic or minimally symptomatic metastatic lesions up to 30 mm im longest diameter, with sum of volumes less than 15 ml (the largest lesion must have less then 30 mm in longest diameter and its volume should be less than 10 ml); SRT doses, specified at 80% isodose: 20 Gy for lesions with diameter equal or less than 20 mm, 18 Gy for 21–30 mm lesions.) PTV will be obtained by adding 2-mm margin to GTV.

Standard WBRT will pe performed for patients who do not qualify for SRT; 30 Gy in 10, 2-Gy fractions will be given in 2 weeks.

### Statistical assumptions

The sample size of the studied group of patients was calculated using the online SWOG data calculator (Southwest Oncology Group). Based on the published data (in 2013) from our cancer center reporting the survival of patients with SCLC treated in our institution in 2003 to 2006, the expected number of patients was calculated. The 2-year survival was 36% for a group of 138 LD-SCLC treated in our institution [17]. We assumed that the 2-year survival in the studied group will be at least 50%. Taking α < 0.1 and a power of 0.80, the required sample size is 80 patients. Patients with diagnosed LD-SCLC, after radical treatment with remission or at least a good response after radical therapy, will be eligible for the study. Patients would have had radical radiotherapy to the chest with chemotherapy according to current international guidelines and institutional protocols. A detailed medical history will be collected regarding SCLC and comorbidities. During the qualification visit, the remission or good response should be confirmed based on chest/abdomen/pelvis CT examinations. After obtaining the patient’s informed consent to participate in the clinical trial, a brain MRI (MRI-1) will be performed and a baseline assessment of cognitive functions will be carried out using the battery of test listed below. Patients who at MRI-1 will be diagnosed with cerebral metastasis, depending on the size and number of lesions, will be eligible for WBRT (whole brain irradiation) or SRT (brain stereotactic radiotherapy). We propose SRT eligibility treatment criteria as in NSCLC according to the standards of the participating centers. All participating radiotherapy centers must have experience in SRT of brain metastases and up-to-date radiotherapy equipment suitable for SRT. Subjects who will not have cerebral metastases in the MRI-1 after chemoradiotherapy will have their next follow-up every 3 months (± 2 weeks) up to 2 years, and then every 6 months (± 2 weeks) up to 3 years. At the qualifying visit before MRI-1, and then every 6 months ±2 weeks), the patients will have a cognitive examination performed using dedicated neuropsychological tests and QoL assessment using the QLQ-C30 questionnaire. The tests will be conducted in the following order: California verbal learning test (CVLT) with a delay of 15 min, Color connection test (CCT), CVLT (after delay), Benton visual retention test (BNRT), verbal fluency test (VFT) by the certified psychologist (Table 2).

**Table 2:** Schedule for observation of patients qualified for the study (24 months)

	initial visit	observation-months									
Months	−1	3	6	9	12	15	18	21	24	30	36
Patient consent	x										
Patient history and physical exam	x	x	x	x	x	x	x	x	x	x	x
Neurocognitive tests	x		x		x				x		
Quality of life	x				x				x		
Brain MRI scan and chest/abdomen CT scan	x	x	x	x	x	x	x	x	x	x	x

Overall survival will be measured using the Kaplan-Meier method from the date of the chest chemoradiotherapy start day until death, with preplanned subgroup analysis of brain metastases-free and brain metastases group. Assessment of clinical response by RECIST 1.1 criteria after brain SRT and WBRT in the brain will be determined. Comparison of neurocognitive tests scores in the subgroup with developed brain metastases before and after SRT and/or WBRT will be carried out using Student’s *t* test or in case of non-normal distribution by nonparametric tests. In the subgroup without developed brain metastases, the analysis of tests scores will be done in the same way. QoL scores and neurocognitive test scores will also be compared between subgroups with and without metastases to the brain with nonparametric tests. The tested differences will be considered significant if *p* value is less than 0.05. In order to consider important prognostic factors and stratification factors related to the disease and the treatment modality in the final analysis, initial tumor volume of intrathoracic disease (GTV), mode of chemoradiation treatment: concurrent vs sequential and bid (irradiation twice daily) vs oid (irradiation once daily) will be recorded in the data base.

## Discussion

The trial design was discussed with several SCLC patients by SN. Patients diagnosed with SCLC in our current clinical practice after being informed about benefits and potential harms of PCI often consciously resign from PCI; therefore, prospective study without control arm with PCI was chosen instead of randomized controlled trial. Participation in the study is completely voluntary, the patient will be informed about the generally recognized standard of therapeutic treatment and the possible consequences of participating in the study. Regular patient controls will be carried out in the presence of experienced specialists, and when the disease progresses, the patient will be treated immediately in an optimal manner according to current clinical practice. Personal data of the subject will be closely guarded. Project documentation will be protected so that undesirable persons will not have access to it. At any time during the project, the patient has the right to resign from further participation.

There are 3 radiotherapy centers that have declared to take part in this study: (1) Szpital MSWiA z Warmińsko-Mazurskim Centrum Onkologii w Olsztynie, (2) Ośrodek Radioterapii i usprawniania NU-MED w Elblągu, (3) Klinika Onkologii i Radioterapii Gdańskiego Uniwersytetu Medycznego w Gdańsku. The planned enrollment time is 24 months. The first patient was enrolled in Szpital MSWiA z Warmińsko-Mazurskim Centrum Onkologii w Olsztynie in September 2019. In case of enrolment rate slower than expected, additional radiotherapy centers in Poland will be approached to participate in the study.

## Data Availability

The data sets used and/or analyzed during the current study are available from the corresponding author on reasonable request.

## Abbreviations

PCI: Prophylactic cranial irradiation
LD-SCLC: Limited disease small cell lung cancer
MRI: Magnetic resonance imaging
SRT: Stereotactic radiotherapy
WBRT: Whole brain radiotherapy
QoL: Quality of life
CNS: Central nervous system
CT: Computed tomography
HR: Hazard ratio
ED-SCLC: Extensive disease small cell lung cancer
RTOG: Radiation Therapy Oncology Group
SWOG: Southwest oncology group
RECIST: Response Evaluation Criteria in Solid Tumors
CVLT: California verbal learning test
CCT: Color connection test
BNRT: Benton retention test
VFT: Verbal fluency test
